# Definition of compartment-based radical surgery in uterine cancer: radical hysterectomy in cervical cancer as ‘total mesometrial resection (TMMR)’ by M Höckel translated to robotic surgery (rTMMR)

**DOI:** 10.1186/1477-7819-11-211

**Published:** 2013-08-26

**Authors:** Rainer Kimmig, Pauline Wimberger, Paul Buderath, Bahriye Aktas, Antonella Iannaccone, Martin Heubner

**Affiliations:** 1 Department of Gynecology and Obstetrics, West German Cancer Center, University Clinic Essen, University of Duisburg-Essen, Hufelandstrasse 55, Essen 45147, Germany; 2 Department of Gynecology and Obstetrics, University Clinic Carl Gustav Carus, Dresden University of Technology, Fetscherstrasse 74, Dresden 01307, Germany

## Abstract

**Background:**

Radical hysterectomy has been developed as a standard treatment in Stage I and II cervical cancers with and without adjuvant therapy. However, there have been several attempts to standardize the technique of radical hysterectomy required for different tumor extension with variable success. Total mesometrial resection as ontogenetic compartment-based oncologic surgery - developed by open surgery - can be standardized identically for all patients with locally defined tumors. It appears to be promising for patients in terms of radicalness as well as complication rates. Robotic surgery may additionally reduce morbidity compared to open surgery. We describe robotically assisted total mesometrial resection (rTMMR) step by step in cervical cancer and present feasibility data from 26 patients.

**Methods:**

Patients (n = 26) with the diagnosis of cervical cancer were included. Patients were treated by robotic total mesometrial resection (rTMMR) and pelvic or pelvic/periaortic robotic therapeutic lymphadenectomy (rtLNE) for FIGO stage IA-IIB cervical cancer.

**Results:**

No transition to open surgery was necessary. No intraoperative complications were noted. The postoperative complication rate was 23%. Within follow-up time (mean: 18 months) we noted one distant but no locoregional recurrence of cervical cancer. There were no deaths from cervical cancer during the observation period.

**Conclusions:**

We conclude that rTMMR and rtLNE is a feasible and safe technique for the treatment of compartment-defined cervical cancer.

## Background

The ontogenetic compartment theory states that malignant tumor growth is confined to permissive compartments derived from a common primordium in embryonic development. Tumor permeation may be facilitated in the permissive ontogenetic compartment but suppressed at the compartment borders [1]. The existence of developmental compartments was shown first in Drosophila [2] and reviewed by Dahmann *et al.*[3]. A clinical implementation was described first for total mesorectal excision (TME) in the treatment of rectal cancer [4,5]. Höckel and Fritsch investigated embryonic development of the female reproductive tract with respect to embryological different compartments [6-8] and were able to define three different primordial tissue complexes from cranial to caudal: the paramesonephric-mesonephric-Müllerian tubercle complex, >the deep urogenital sinus (UGS) vaginal plate complex and the superficial UGS-genital folds and tubercle process [1]. First evidence for the functionality of this theory concerning the paramesonephric-mesonephric-Müllerian tubercle complex in cervical cancer has been shown for cervical cancer with respect to local tumor control following total mesometrial resection (TMMR) without any adjuvant radiotherapy [9,10] but also with respect to the pattern analysis of local tumor spread in advanced and recurrent disease [11]. It has to be questioned whether the confirmation of these findings could fundamentally change the classification of radical hysterectomy and the indication for adjuvant radiotherapy [12].

With respect to regional spread data and pelvic therapeutic lymphadenectomy (tLNE), they were also systematically analyzed and also assigned to ontogenetic lymphatic compartments, which may be classified as external iliac nodes, paravisceral nodes, common iliac nodes and presacral nodes [13].

The original technique has been developed and described in open surgery. At present, increasing numbers of minimally invasive approaches to radical surgery in cervical cancer are being reported. However, it is difficult to compare the results while lacking a systematic description of the different techniques used. The TMMR and tLNE may be perfectly standardized, but the technique of robotically assisted minimally invasive preparation may differ markedly to the open approach. We recently reported on the technique of therapeutic pelvic and paraaortic lymphadenectomy transferred to robotic surgery in genital cancer with special respect to uterine cancer. Therapeutic lymphadenectomy is performed for control of regional tumor spread; dependent on the original site of the primary tumor, however, it has mandatorily to be combined with complete removal of the appropriate local compartment at risk. In the case of cervical cancer, the removal of the local compartment can be performed as a total mesometrial resection as already outlined. This technique has been translated to robotically assisted laparoscopic surgery (rTMMR) with the support of Michael Höckel. To define this procedure reproducibly with respect to anatomical landmarks, we describe the technique step by step and present feasibility data from the first 26 patients. We present this concept of rTMMR internationally (the technical principles without feasibility data were reported up to now in German only and, therefore, are not accessible to the majority of international oncological surgeons [14]) intending to provoke scientific discussion within the community of surgical oncologists. This discussion should primarily take place with respect to the impact of robotically assisted compartment-adapted radical hysterectomy in cervical cancer; however, it also demonstrates the impact of exact visual definition of crucial surgical steps for future research in surgical oncology and comparison of clinical data.

## Methods

### Surgical technique

The first author has been trained in the surgical technique of TMMR and tLNE, attending in the Leipzig School of Surgery in 2006. All radical hysterectomies were consequently performed using the ontogenetically derived, nerve-sparing technique of TMMR and tLNE, if adequate. In 2010, robotically assisted laparoscopic surgery was implemented using a da Vinci™ Surgical System (Intuitive Surgical Inc., Sunnyvale, CA, USA). The principles of the surgical steps were systematically translated to robotic surgery and optimized to guarantee the same radicalness compared to open surgery but preserving the advantages of an endoscopic approach. The different steps were discussed with M. Höckel by video sequences. Finally, M. Höckel participated in the surgery in Essen and confirmed the equality of the robotic approach with respect to his operation technique defined by open surgery. The resulting rTMMR technique will be described in detail.

**Figure 1:**
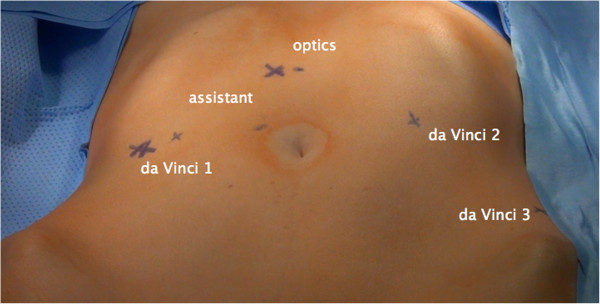
**Positioning of trocars for rTMMR and rtLNE in cervical cancer.** rtLNE, robotic therapeutic lymphadenectomy; rTMMR, robotic total mesometrial resection.

Preparation of the patient: identical to laparoscopy, Trendelenburg positioning of at least 25 to 30 degrees. Side docking of the patient cart. Positioning of the trocars: camera trocar 10 cm to 15 cm supraumbilically (that is 25 cm above the symphysis), two lateral robotic trocars about 5 cm to 10 cm above the upper anterior iliac spine on both sides, that is one additional robotic trocar on the left between the camera trocar and left lateral trocar, one assistant trocar of 10 mm diameter on the right side between the camera trocar and right lateral trocar. The space in between the trocar incisions should be at least 10 cm to ensure free adequate mobility (Figure 1).

### Patients and specimens

As a first proof of feasibility, 26 patients with cervical cancer FIGO stage IA-IIB were treated by robotic total mesometrial resection (rTMMR) +/− robotic therapeutic lymphadenectomy (rtLNE). All patients gave their informed consent to the procedure. Therapeutic lymphadenectomy was performed by robotic surgery in analogy to the procedure described by M. Höckel [6,9]. The complete clearance of the lymph node basins of the lymphatic drainage system - including the intercalated mesometrial nodes and the lymph basins - downstream to the Müllerian compartment was defined to be a superior quality parameter compared to the number of removed lymph nodes as described previously by the authors [15]. The lymph node basins for cervical cancer include: paravisceral nodes (internal iliac nodes including gluteal and rectal nodes, (pv)), external iliac nodes (ei) first line, and common iliac nodes (ci) and presacral/subaortic nodes (ps) on both sides, second line. Periaortic nodes including inframesenteric (im) and, if indicated, supramesenteric/infrarenal (sm/ir) nodes were removed in case of pelvic nodal disease, since these basins have to be considered tertiary and quaternary with respect to the cervix. Perioperative morbidity and early postoperative morbidity were analyzed. In addition, we noted perioperative blood loss by hemoglobin levels and frequency of perioperative blood transfusions. The tumor-related outcome was recorded.

### Statistical analysis

Analysis of clinical and histopathological data was performed using SPSS version 17.0 for Macintosh™ (SPSS, Chicago, IL, USA). We conducted a descriptive analysis only, considering the limited number of patients and the explorative character of this analysis.

### Technique and results

rTMMR with tLNE were performed in a steep Trendelenburg position, trocar positioning is depicted in Figure 1. Prior to rTMMR, therapeutic lymphadenectomy starting at the aortic bifurcation has been performed as specified previously [15]. Accordingly, all regional nodes of lymph node basins at risk are removed, except for intercalated mesometrial nodes located predominantly in the vascular mesometrium.

**Figure 2:**
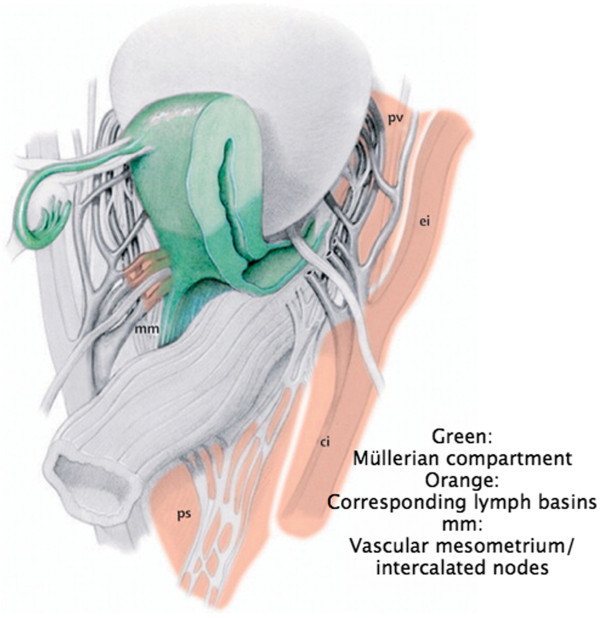
**The structures of the female genital tract with reference to the embryologic Müllerian compartment (green) and the corresponding primary and secondary lymph basins (from Höckel *et al*., Resection of the embryologically defined uterovaginal (Müllerian) compartment and pelvic control in patients with cervical cancer: a prospective analysis. ***Lancet Oncol* 2009, with permission from ELSEVIER).

Prior to the standardized description of rTMMR, the principles and nomenclature of M. Höckel’s method should be commemorated. As shown in Figure 2, the Müllerian compartment consists of the uterus, the fallopian tubes, the vascular mesometria and the fibrofatty (ligamentous) mesometria (mesocolpia) and the proximal vagina. The vascular mesometria correspond to the tissue surrounding and accompanying the uterine vessels to the iliac vessels and along their anastomoses to the vesical vessel system anteriorly. The fibrofatty (ligamentous) mesometria (mesocolpia) correspond to the sacrouterine and rectouterine/vaginal ligaments posteriorly, which insert dorsally/laterally along the pubo- and ileococcygeus muscles and medially at the mesorectum and the rectovaginal septum. Thus, this structure forms a complex geometry of a half-cylinder sagittally curved according to the pelvic axis. All these structures have to be removed completely. Only the vagina, also belonging to the Müllerian system with its upper two-thirds, should not be removed completely for functional reasons. Consequently, the resection at the level of the vagina should have wide clear margins confirmed by histology. With respect to all other resection borders, clear margins are achieved by removal of the total Müllerian compartment irrespective of the distance to the resection border as long as the tumor does not transgress the compartment.

The removal of the uterus, the fallopian tubes, the vascular and ligamentous mesometria and the part of vagina starts dorsally.

**Figure 3:**
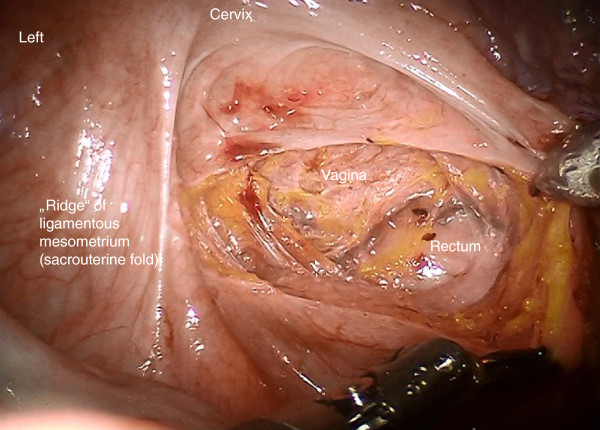
Incision of the pouch of Douglas to develop the rectovaginal space and prepare the medial aspect of ligamentous mesometria (rectovaginal ligaments).

Step 1. First, the peritoneum will be incised pararectally to the pouch of Douglas from right to left and the rectum is mobilized medially from the vaginal wall (Figure 3). Rectovaginal and rectouterine ligaments that form the medial part of the ligamentous mesometrium are lateralized until their insertion into the perirectal tissue is reached.

**Figure 4:**
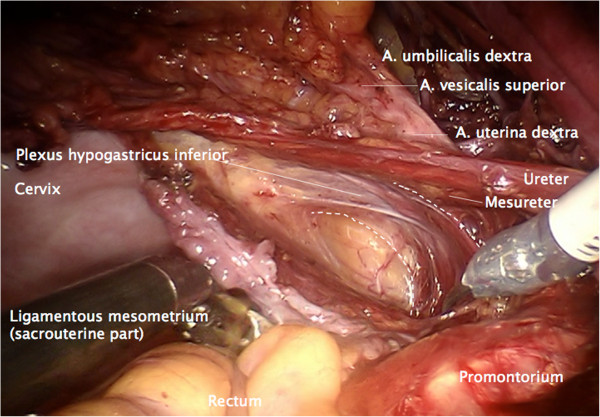
**Preparation of the lateral part of ligamentous mesometria demonstrated on the right.** Developing of the avascular space between the ureter, the mesureter and the inferior hypogastric plexus laterally/ventrally and the ligamentous mesometrium medially/dorsally (the sacrouterine ligament).

Step 2. Now, the ureter is identified and prepared in continuation with the mesureter and the adjacent inferior hypogastric plexus and nerve. Thus, the avascular plain between the lateral part of the ligamentous mesometrium (that is the sacrouterine ligament) may easily be opened and the nerve plain can be dissociated laterally from the mesometrium (Figure 4).

**Figure 5:**
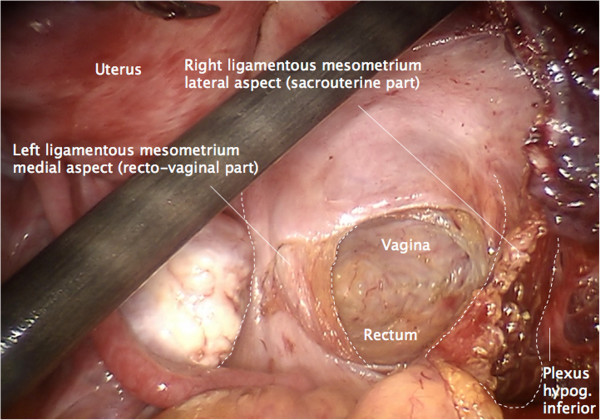
Overview following the preparation of the ligamentous mesometria with simultaneous demonstration of the medial (rectovaginal, on the left) and the lateral aspect (sacrouterine, on the right).

**Figure 6:**
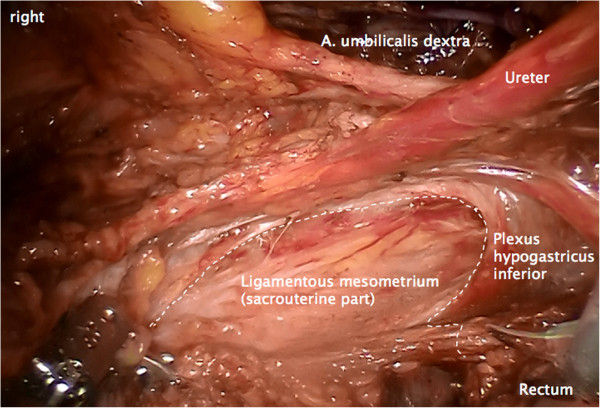
Lateral resection line of the ligamentous mesometrium on the right starting from the ‘ridge’ pararectally, following the dorsal/lateral rim of the inferior hypogastric plexus to the insertion along the iliococcygeal and pubococcygeal muscle and the fascia endopelvina.

**Figure 7:**
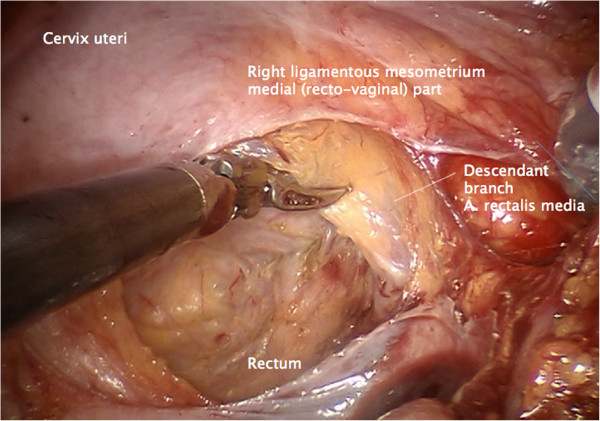
Medial resection of the ligamentous mesometrium on the right starting from the ‘ridge’ dissecting the connective tissue junction along the descendant branch of the rectal artery to separate the mesorectum.

**Figure 8:**
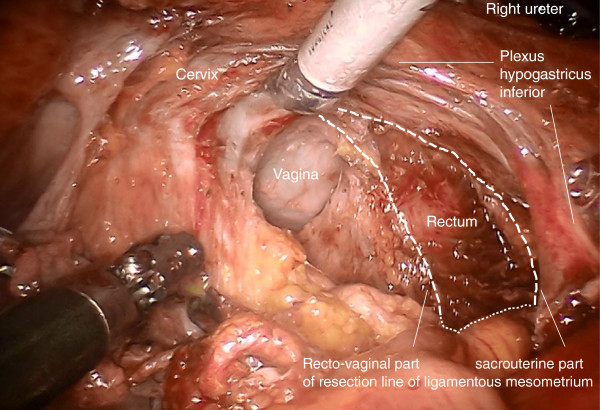
Topography demonstrating complete resection of the ligamentous mesometrium on the right keeping the mesorectum and the inferior hypogastric plexus intact.

**Figure 9:**
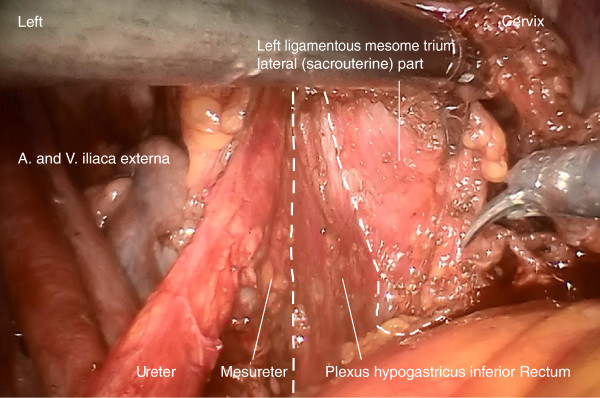
Lateral resection line of the ligamentous mesometrium on the left during resection.

Step 3. The medial and lateral part of the ligamentous mesometrium may now be completely exposed (Figure 5) and resected first laterally, pararectally to the pelvic wall (the sacrouterine part, along the coccygeus and iliococcygeus muscle and the endopelvic fascia, Figure 6) and then, prerectally along the descending branch of the rectal artery (the rectouterine/vaginal part, Figure 7), starting on the right (resection lines, Figure 8), finalizing on the left (Figure 9).

Step 4. To detach the vesicouterine attachment, the anterior surface of the uterus has to be exposed and put under tension to facilitate incision of the peritoneum at the vesicouterine fold. The peritoneum has to be incised in the direction of the lateral part of the round ligaments to divide these at the entrance to the inguinal channel. The loose connective tissue is dissected until the ureters are identified entering the vesical wall (Figure 9). The preparation is extended laterally and the border of the vesical and Müllerian compartments is identified.

**Figure 10:**
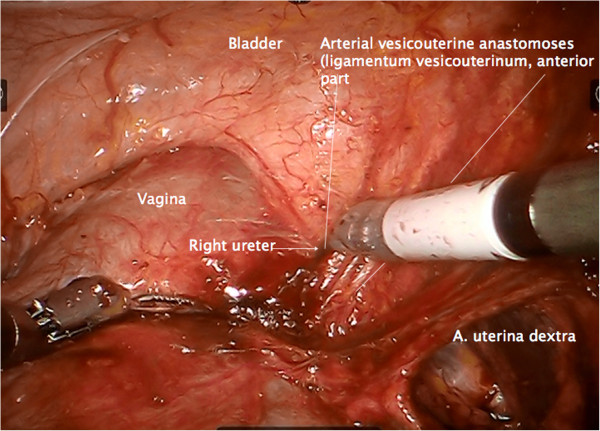
Separation of the Müllerian compartment from the bladder compartment anteriorly, preparing the ureteral entrance to the bladder wall from medially exposing the vesicouterine arterial vessels connecting the Müllerian with the bladder compartment (the anterior part of the vesicouterine ligament) shown on the right.

Step 5. Now, the umbilical artery is identified and completely prepared to its origin from the internal iliac artery. Thus, branching of the uterine artery and one or more superior vesical arteries can be easily identified (Figure 10).

**Figure 11:**
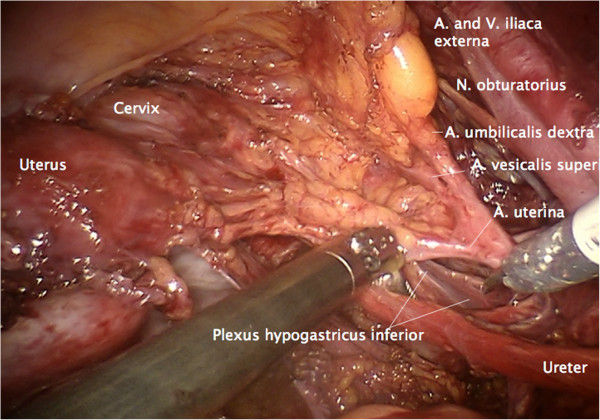
Preparation of the vascular mesometrium (the upper ridge marked by the uterine artery) on the right dissecting the avascular plane dorsally with separation of the ureter, the mesureter and the inferior hypogastric plexus dorsally/medially.

**Figure 12:**
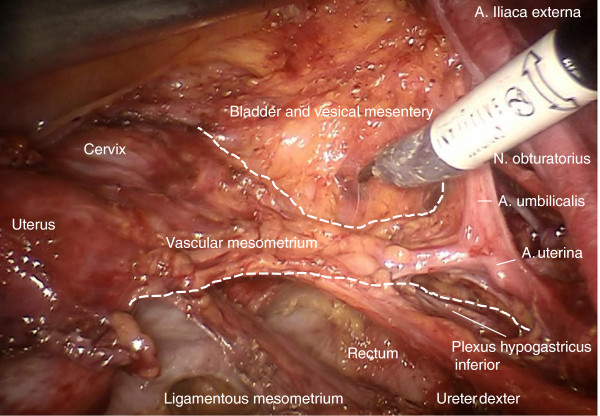
Dissection of the vesical compartment (the proximal bladder mesentery) from the anterior surface of the vascular mesometrium by separating the bordering lamella of the two compartments on the right to prepare its complete resection containing blood and lymphatic vessels and intercalated nodes.

Step 6. At this time, the vascular mesometrium can be exposed, first, by developing the avascular space between its anterior surface and the bladder mesentery containing the superior vesical artery (Figure 11) second, by developing the avascular space between the posterior surface and the plain of the ureter, mesureter and hypogastric plexus (Figure 12). The caudal border of the vascular mesometrium is marked by the deep uterine vein, which should also be resected.

**Figure 13:**
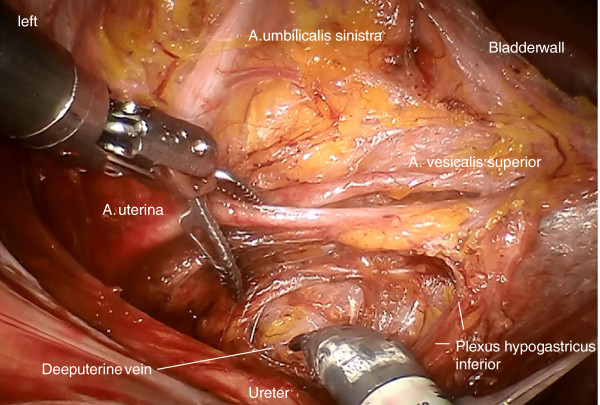
Identification of the deep uterine vein and dissection of vascular mesometrium at the level of the origin from internal iliac vessels shown on the left.

Step 7. The complete resection of the vascular mesometrium including all paracervical/mesometrial intercalated nodes starts with the coagulation and division of the uterine artery and superficial veins at their origin from the iliac vessels. This step should be carried out without separating the vessels in order to remove all perivascular lymphatic tissue. The deep uterine vein is identified and coagulated separately (Figure 13).

**Figure 14:**
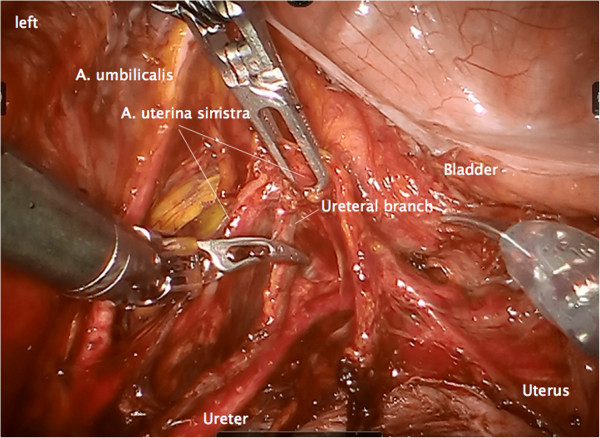
Elevation of the dissected vascular mesometrium and dissection of the ureteral branches of the uterine vessels to separate the ureter from the vascular mesometrium on the left.

**Figure 15:**
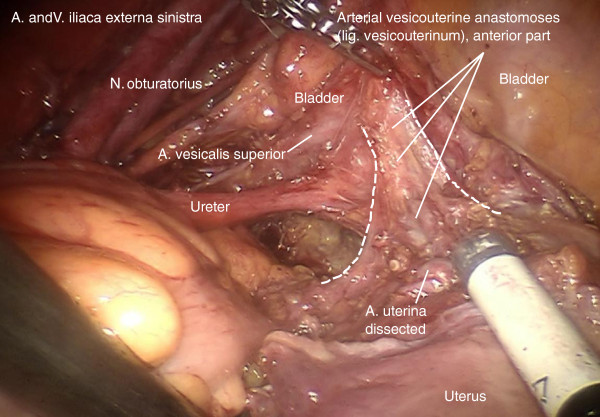
**The vascular mesometrium can now be flipped dorsally without elevating the ureter and the vesicouterine vascular junction can be exposed from laterally on the left (the vesicouterine ligament).** The connecting vessels and the accompanying connective tissue are dissected at their free part close to their junction to the bladder vessels.

Step 8. Dissection of the ureteral branch of the uterine artery (and vein) following elevation of the uterine bundle to mobilize the ureter in its ‘tunnel’ (Figure 14). Coagulation and dissection of vesicouterine/vaginal arterial anastomoses in the anterior part of vesicouterine ligament to separate the Müllerian from the vesical compartment ventrally, carefully preserving the ureteral branches of the bladder mesentery (Figure 15).

**Figure 16:**
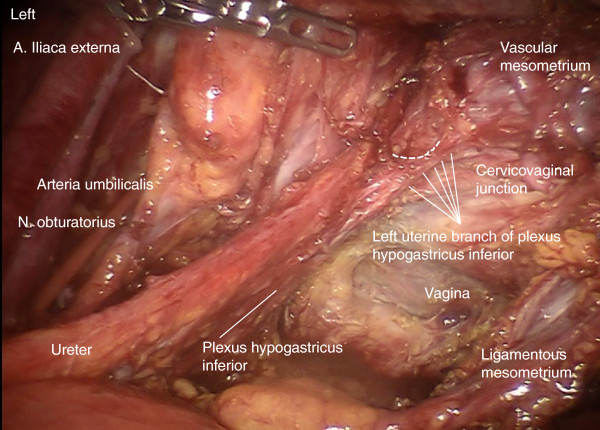
To separate the inferior hypogastric plexus, the mesureter and the ureter en bloc from the Müllerian compartment, the uterus-supplying nerve fibers have to be dissected at their branching laterodorsally of the cervix as shown on the left.

Step 9. Dissection of the uterine nerve fibers at the lateral posterior aspect of the cervix (Figure 16). The inferior hypogastric vesical nerve branches can then be mobilized and pushed down laterally together with the ureter to preserve them.

**Figure 17:**
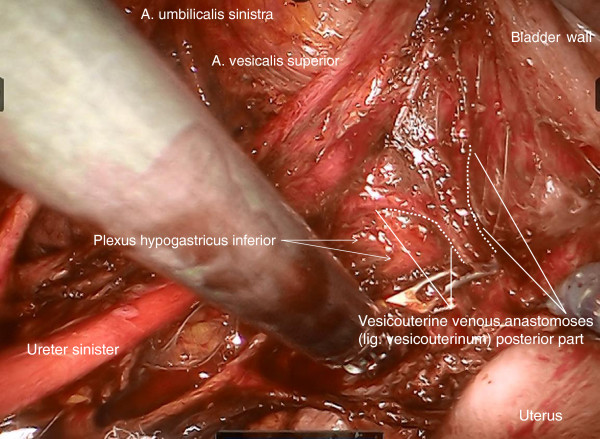
Finally, the vesicouterine/vesicovaginal venous anastomoses dorsally to the ureter representing the posterior part of vesicouterine ligament have to be dissected to complete the dissection of the vascular mesometrium as shown on the left.

Step 10. Dissection of the ‘ladder-like’ arranged vesicovaginal venous anastomoses (Figure 17), if not already done during the separation of the ventral aspect of the vascular mesometrium from the bladder mesentery (the so-called ‘posterior leaf of the vesicouterine ligament’).

**Figure 18:**
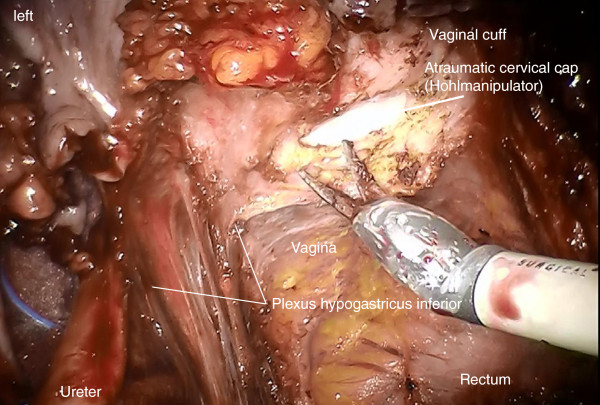
**Following the definition of the vaginal resection line and adequate preparation of the mesocolpium, the incision of the vaginal wall starts dorsally on the left and will be continued dorsally to the right following the right side wall to the anterior wall ending up again on the left.** Tumor exposition has to be avoided during removal of the specimen.

Step 11. Definition of the vaginal resection plane and dissection of the paracolpium, if necessary. Opening the vagina dorsally and the resection of the vaginal cuff with sufficiently clear margins confirmed by frozen section (Figure 18).

**Figure 19:**
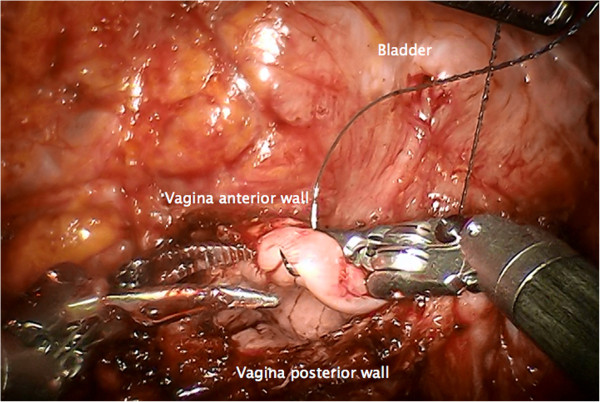
**Closure of the vagina by running suture.** A tight closure of the lateral angles can avoid the development of vaginal vault dehiscense.

Step 12. Removal of resected tissue, bags and sponges, and confirmation of clear margins. The vagina is closed by running suture (Figure 19).

**Figure 20:**
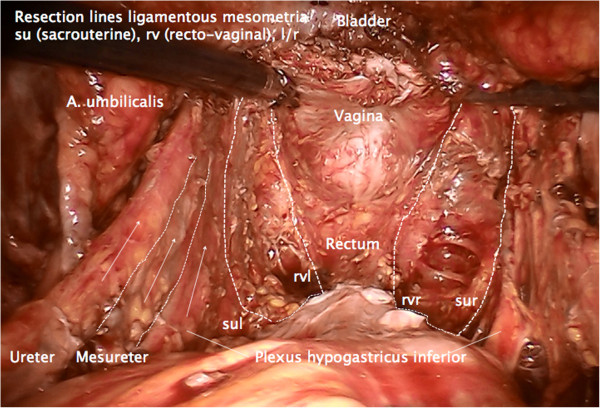
**Pelvic topography following TMMR demonstrating complete resection of the Müllerian compartment including the vascular and ligamentous mesometria except for the retained part of the vagina.** It is clearly visible that the plane of the ureter, mesureter and inferior hypogastric nerve plexus are completely intact on both sides. TMMR, total mesometrial resection.

Finally, all tissue of the Müllerian compartment has been removed completely, but adjacent structures such as the ureter, mesureter, hypogastric plexus, rectal and vesical mesentery are entirely preserved (Figure 20). Thus, this procedure may be performed identically for all compartment-defined cervical cancers ensuring maximum radicalness with respect to tumor resection without increasing morbidity by extending radicalness to neighboring structures. The adaption of surgery to compartments instead of tumor size and extent prevents unnecessary morbidity.

In contrast to M. Höckel, we do not readapt the natural sigma adhesions, representing the sole difference to the original method described for open surgery. However, this may be easily implemented if desired.

## Results

In total, 26 patients with the diagnosis of cervical cancer underwent surgery. All patients received a total mesometrial resection of the uterus. Therapeutic pelvic and, if necessary, periaortic lymphadenectomy was added when indicated.

Three patients had been treated by radiochemotherapy first due to positive periaortic nodes and/or local inoperability. In these patients, TMMR was performed as ‘adjuvant’ treatment after completion of radiochemotherapy. In all other patients, surgery was the primary treatment. The mean age of the patients was 49.5 years (31 to 75 years). The mean body mass index (BMI) of the patients was 24.3 kg/m^2^ (range 18 to 33 kg/m^2^), 42% had preoperative comorbidity and prior intraabdominal surgery was noted in 54%.

The distribution of the FIGO stages and surgical procedures can be seen in Table 1. There were 20/26 squamous cell cancers (77%) and 6/26 adenocarcinomas. Positive nodes were detected in 5/23 (22%) patients with primary surgery, whereas one of the patients who had primary radiochemotherapy had positive periaortic nodes at pretherapeutic staging (16/36). Frequency of high grade tumors (G3) was 5/23 (22%) and 5/23 (22%) tumors showed lymphangioinvasion (L1).

**Table 1:** Correlation of tumor stage, preoperative treatment, lymph node count and complication rate with type of surgical procedure

	**Patients**	**FIGO Stage**			**Post radiochemo-therapy**	**Blood transfusion**	**Complication rate**	**Mean lymph node count**
		**IA**	**IB**	**II A/B**				
**TMMR only**	6	3	0	0	3	0	1	-
**TMMR and pelvic LNE**	15	4	10	1	0	1	4	32.4
**TMMR, pelvic and pa LNE**	5	0	4	1*	0	3	1	42.2
**Total**	26	7	14	2	3	4	6	34.9

*And partial colpectomy. *TMMR* total mesometrial resection, *pa LNE* periaortic lymphadenectomy.

All interventions were performed as intended without modifications and no transition to open surgery was necessary due to complications or technical problems. Tumor resection was microscopically confirmed being complete (R_0_) in all cases. Mean lymph node count was 32.4 for pelvic and 42.2 for pelvic and periaortic lymphadenectomy, respectively. Periaortic lymphadenectomy was performed inframesenterically only in the case of proven negative periaortic nodes. In any case, it was not the number of nodes, but video-documented, complete clearance of lymph node basins that was taken as criterion for sufficient therapeutic lymphadenectomy as outlined in [13].

There were no intraoperative complications, in total postoperative complications occurred in six patients (23%). Two had minor local wound infection, two infected lymph cysts requiring revision, one had a postoperative laparoscopic revision for bleeding and one patient had a vaginal cuff dehiscence.

With respect to blood loss, hemoglobin levels were determined pre- and postoperatively (on the first day). Mean decrease of hemoglobin concentration was determined to be 2.1 g/dl in TMMR (0.5 to 3 g/dl), 3.3 g/dl in TMMR and pelvic lymphadenectomy (1.1 to 4.9 g/dl) and 3.3 g/dl in TMMR and pelvic/periaortic lymphadenectomy (2.4 to 4.6 g/dl). Blood transfusion was applied in four patients (15%) showing postoperative hemoglobin levels of 7.8, 8.3, 8.6 and 10.3 g/dl. Mean follow-up of the patients was 18 months (range 2 to 27 months). One patient who had received primary radiochemotherapy was lost to follow-up. There was one distant recurrence (1/25 corresponding to 4%). One intercurrent death occurred at 18 months postoperatively without evidence of tumor recurrence or association to cancer therapy. No patient has died of tumor or sequelae of the tumor-associated therapy during the observation period.

The patient with recurrence presented with stage pT2b, pN1, G2 and had a rupture of the anterior cervical fascia during surgery with exposure of the tumor to the surgical field. She refused radiation therapy and developed trocar site recurrence after 9 months, treated by radiation therapy and surgical excision, respectively. There was no additional locoregional recurrence. At evaluation, 20 months after diagnosis of recurrence, she was doing well without evidence of disease.

## Discussion

We were able to show that the principle of compartment-based surgery performed as total mesometrial resection (TMMR) combined with therapeutic lymphadenectomy (tLNE) as described by M. Höckel [9,10] can systematically be translated to minimally invasive, robotically assisted procedures (rTMMR and rtLNE). Robotic surgery enables us to develop and dissect structures with high ‘optical’ accuracy, thus allowing us to prepare and remove compartment-associated tissue completely without injuring adjacent structures by respecting the filmy septa at the compartment borders; thus, excellent visual documentation by HD video recording is dramatically facilitated.

The method appears to be feasible and safe. It has to be considered that the presented data were collected consecutively during the first attempts in this surgical procedure. However, the mean follow-up time of 18 months is limited and the number of patients (with respect to the fact that six patients at FIGO stage IA and three patients who had prior radiotherapy were included) is admittedly small, it should be noticed, nevertheless that there has been no locoregional tumor recurrence during this observation period. Thus, it may be assumed that laparoscopic, robotically assisted TMMR and tLNE (rTMMR and rtLNE) may be safe with respect to local tumor control. This would be in accordance with the impression that due to the excellent 3D vision, along with the magnification of the surgical field and the high precision and control of movements, the accuracy of this technique may be considered at least equivalent to open surgery. Therefore it may be assumed that a comparable radicalness may be achieved.

As a consequence, further development of minimally invasive surgical techniques for treatment for cervical cancer confined to the Müllerian compartment should focus on evaluation of rTMMR and rtLNE. First, compartment-based surgery appears to exert excellent locoregional control in cervical cancer combined with a low complication rate as shown for open surgery [10,13]. Second, the translation to a minimally invasive method by robotic assistance adds the advantages of minimally invasive surgery regarding blood loss, mobilization, length of hospitalization and short-term complications as it has previously been shown for robotic surgery [16,17]. With respect to the port-site recurrence in our report, we stress that exposure of the tumor to the surgical field must strictly be avoided. Although port-site metastases are a well-known phenomenon, they seem to be a rare event [18,19]. In robotic surgery, it is reported that the rate of port-site metastases is low and similar to conventional laparoscopy [20,21]. Nevertheless, we recommend ensuring that no direct contact of the tumor with the operative field takes place. In this regard, we consider the closure of the cervical channel or the vagina prior to the opening of the vagina abdominally an important procedure in uterine cancers.

## Conclusions

In conclusion, we suggest that the minimally invasive approach of compartment-based oncologic surgery for uterine cancers by robotic assistance is feasible, safe and may be beneficial for patients with cervical cancer confined to the Müllerian compartment. In order to evaluate whether the excellent monocentric data with outstanding locoregional tumor control and low morbidity of M. Höckel in cervical cancer [6,9,10] holds true in a multicentric setting, an observational study has been initiated. Starting recruitment in 2013, results of TMMR and tLNE with respect to morbidity and survival will be analyzed. Participation will be independent of surgical access - open or laparoscopic or laparoscopic robotically assisted - but requires verifiable standardization with an accordant training of TMMR, tLNE and pathological workup. For robotically assisted total mesometrial resection (rTMMR) the technique described in this publication will be the reference basis. Interested experienced surgeons who are willing to fulfill the requirements are invited by the first author to apply for participation.

In future, from our point of view, this kind of preparation and auditing of surgical studies could contribute to a better comparability between different sites and surgeons, which is not only mandatory in scientific studies, but may also beneficial in education and clinical practice. The clear definition of a surgical procedure, supported by educational illustrations and videos may serve as a pioneer of a new generation of surgical studies and - not least - surgical education.

## Competing interests

The manuscript has been approved by all authors. We declare that there are no conflicts of interest. Rainer Kimmig has received expense allowances/honoraria from Intuitive Surgical Inc. in 2011 to 2013 for educational work in robotic surgery and training of gynecologic departments in Western Europe (proctoring).

## Authors’ contributions

The authors all made substantial contributions. RK contributed to the conception and design, analysis and interpretation of data, drafting and finalization of the manuscript and conceiving the analysis. PW contributed to the conception, acquisition of data and critically revising the manuscript. PB contributed to the acquisition of data and drafting the manuscript. BA contributed to the acquisition and analysis of data and critically revising the manuscript. AI contributed to the acquisition and statistical analysis of data and drafting the manuscript. MH contributed to the design, data interpretation, critical revision of the manuscript and drafting the manuscript. All authors read and approved the final manuscript.
